# Priming the Secure Attachment Schema Affects the Emotional Face Processing Bias in Attachment Anxiety: An fMRI Research

**DOI:** 10.3389/fpsyg.2017.00624

**Published:** 2017-04-20

**Authors:** Qingting Tang, Xu Chen, Jia Hu, Ying Liu

**Affiliations:** School of Psychology, Southwest UniversityChongqing, China

**Keywords:** attachment style, attachment anxiety, secure base schema, emotional face processing bias, fMRI

## Abstract

Our study explored how priming with a secure base schema affects the processing of emotional facial stimuli in individuals with attachment anxiety. We enrolled 42 undergraduate students between 18 and 27 years of age, and divided them into two groups: attachment anxiety and attachment secure. All participants were primed under two conditions, the secure priming using references to the partner, and neutral priming using neutral references. We performed repeated attachment security priming combined with a dual-task paradigm and functional magnetic resonance imaging. Participants’ reaction times in terms of responding to the facial stimuli were also measured. Attachment security priming can facilitate an individual’s processing of positive emotional faces; for instance, the presentation of the partner’s name was associated with stronger activities in a wide range of brain regions and faster reaction times for positive facial expressions in the subjects. The current finding of higher activity in the left-hemisphere regions for secure priming rather than neutral priming is consistent with the prediction that attachment security priming triggers the spread of the activation of a positive emotional state. However, the difference in brain activity during processing of both, positive and negative emotional facial stimuli between the two priming conditions appeared in the attachment anxiety group alone. This study indicates that the effect of attachment secure priming on the processing of emotional facial stimuli could be mediated by chronic attachment anxiety. In addition, it highlights the association between higher-order processes of the attachment system (secure attachment schema priming) and early-stage information processing system (attention), given the increased attention toward the effects of secure base schema on the processing of emotion- and attachment-related information among the insecure population. Thus, the following study has applications in providing directions for clinical treatment of mood disorders in attachment anxiety.

## Introduction

According to Bowlby’s (1969, 1982) attachment theory, human beings are innately predisposed to establish effective bonds and maintain proximity with their caregiver, from here on referred to as the attachment figure, who provides warmth, nutrition, and protection, all of which are vital for an infant’s survival (Bowlby, 1973; Landers and Sullivan, 2012). Internal working models that form mainly through interactions with the primary caregivers during childhood are referred to as a type of relational schema consisting of specific beliefs about the self and the attachment figure. The categories of attachment styles are now described through a two-dimensional model (Brennan et al., 1998), according to which attachment security corresponds to low avoidance and anxiety scores, reliable social interactions with attachment figures, and positive views of the self and others. Conversely, individuals with attachment anxiety score higher on the attachment anxiety dimension, (Mikulincer et al., 2003) display a preference for seeking acceptance from and proximity with others, fear rejection and abandonment, and have a negative self-image along with a positive other-image. These images, which were called internal working models that formed mainly through vocal and facial interactions with the nurturer, can affect not only the way in which people think, feel, and behave in close relationships but also their emotional information processing (Pietromonaco and Barrett, 2000), especially the processing of facial expressions, which was considered to be an essential medium of communication in early childhood interactions (Bowlby, 1973).

In the last couple of decades, research in the field of attachment has furthered the conceptualization of internal working models by proposing attachment anxiety as an important dimension underlying individual differences in attachment orientation (Brennan et al., 1998; Mikulincer and Shaver, 2003). Highly anxious individuals are thought to activate their attachment behavioral system faster; thus, they show a high emotional reactivity and a tendency for hypervigilance toward emotional and social stimuli (Bartholomew and Horowitz, 1991; Mikulincer and Shaver, 2003, 2007; Pietromonaco et al., 2006), summarized as “hyperactivation” of the attachment system (Cassidy and Kobak, 1988). This hyperactivation is initiated by the individual’s confusion about the viability of seeking proximity when they need help from their attachment figures, and are impacted extensively by their internal working models. As crucial processes to extracting information from the environment, attention mechanisms are considered highly relevant to attachment-related differences in the processing of emotional stimuli, especially potentially threatening stimuli (Fraley et al., 2000). Attention-based paradigms suggest that individuals with high attachment anxiety display increased vigilance toward and a deeper processing of general threat (Ein-Dor et al., 2011; Silva et al., 2012). Several behavioral research groups have provided evidence that attachment anxiety is associated with a tendency for hypervigilance toward emotional stimuli such as emotional facial expressions (Niedenthal et al., 2002; Chris Fraley et al., 2006), and words associated with threat (Mikulincer et al., 2004). In addition, recent investigations have used techniques like functional magnetic resonance imaging (fMRI) and event-related potentials (ERP) to examine how attachment anxiety modulates the processing of emotional information; for example, studies using ERP techniques have demonstrated that anxious individuals have heightened electrophysiological responses as early as 100–300 ms after the presentation of rejection cues or facial expressions, unlike avoidant individuals (Zhang et al., 2008; Zayas et al., 2009). Studies, although a few in number, suggest that higher anxiety scores correlate with stronger reactions in the amygdala to negative social stimuli such as angry faces or negative verbal scenarios (Lemche et al., 2006; Vrtička et al., 2008); amygdala is the brain region associated with rapid environmental threat detection. Moreover, Vrtička et al. (2011) reported that individuals with high anxiety demonstrate differential increases in parahippocampal cortex and activity in amygdala in response to both positive and negative social situations during spontaneous emotional judgments task. Another study investigating the effect of attachment style on brain activities during a simulated experience of social exclusion reported that high anxiety was positively correlated with activity in the insular and anterior cingulate cortex (DeWall et al., 2012). Thus, evidence from neuroimaging research suggests that attachment anxiety is associated with stronger activity not only in response to criticism or rejection but also to positive social signals. However, studies reported in the literature are not entirely consistent. For example, it has been observed that both attachment avoidance and anxiety orient attention away from negative social signals (Dewitte et al., 2007; Dewitte and De Houwer, 2008), whereas other reports indicate early vigilance in the perception of positive and negative facial expressions for both anxious and avoidant attached individuals (Niedenthal et al., 2002). Studies mentioned above have focused mostly on possible relationships between insecure attachment orientations and cognitive processes such as attention and many of them were unable to confirm the assumed differences in attentional processing as a function of the specific type of attachment insecurity. However, despite inconsistencies, these findings suggest that attachment anxiety affects attentional processing of emotional stimuli. Bias in processing emotional information can be an important risk factor for emotional disorders. Most importantly, we must understand the protective mechanisms underlying the attachment working model of low anxiety individuals (secure individuals), as our knowledge of this is currently limited.

Attachment security is an important source of resilience that can help individuals with insecure attachments to prevent mental disorders. Over the last few decades, numerous studies have provided evidence supporting Bowlby’s (1969) claims about the favorable influences of secure attachment (e.g., Mikulincer and Shaver, 2001), such as lower levels of physical arousal under high-pressure situations (Mikulincer and Florian, 1998), a more active self-image (Bartholomew and Horowitz, 1991), and more flexible cognitive structures that help in processing new information more easily (Mikulincer, 1997). The cognitive activation of the secure base schema can lead insecure individuals to display behaviors similar to those of secure individuals, such as more continual positive self-evaluation (Baldwin, 1994), responding more positively to others’ needs (Mikulincer et al., 2001), greater compassion for and willingness to support people needing help (Mikulincer et al., 2005), enhanced ability to solve creative problems (Mikulincer et al., 2011), increased authenticity, tendency to cheat less (Gillath et al., 2010), and less likelihood of experiencing regret in past or current intimate relationships (Schoemann et al., 2012). Reminding insecure individuals of their secure attachment partners, can attenuate neural responses to social exclusion, even in the physical absence of the partners.

As discussed above, there is robust evidence supporting the view that activation of secure base schema can positively influence the well-being of individuals and relationships. However, research examining the effects of activating attachment security on attachment-related and emotional information is limited. Previous research has mostly focused on the possible associations between chronically insecure working models and cognitive processes such as attention and retrieval of attachment-related information (Fraley et al., 2000; Edelstein, 2006). There is only one study reporting that activating attachment security may influence the processing of attachment-related and emotional information (Andriopoulos and Kafetsios, 2015). This study supported the viewpoint that the activation of the secure attachment schema combined with chronic insecure attachment orientations affected the processing of both positive and negative emotional information unrelated to attachment. This finding initially inspired us to explore the protective mechanisms surrounding the activation of secure base schema in individuals with chronically insecure attachment and attachment anxiety in the current study. This represents a “push-pull” mechanism, whereby, insecure attachment orientation is known to affect the processing of emotional expressions; however, priming with a secure base schema plays a beneficial role in this process. Specifically, activating the sense of attachment security by priming the secure base schema results in decreased activity in the brain regions associated with negative emotions, as well as regions associated with “mentalizing” and social judgment and activates a specific pathway of the reward system of the brain at the same time (Bartels and Zeki, 2000). To our knowledge, there has been no fMRI study on the automatic brain responses during an emotional processing task performed after priming with either a secure base or neutral attachment schema as a function of the attachment anxiety dimension. Therefore, the purpose of our study was to examine the priming effects of secure base schema on the processing of emotional stimuli while exploring the interaction of this priming with attachment orientations, and the underlying neural mechanisms.

Thus, in priming with a secure base schema, we can suppose that high anxiety is associated with the activation of a specific pathway of the reward system in the brain, as well as a deactivation in the regions that are responsible for negative emotions and critical social assessment during the processing of negative emotional faces. For positive emotional faces, considering the spreading effects of activation of secure base schema, it was assumed that both levels (high and low) of attachment anxiety are associated with increased activities in brain circuits that are responsible for positive emotions and the regions belonging to the reward system. Because of the associated sensitivity to potential rejection and a strong desire for closeness, anxious attachment, rather than secure attachment, should trigger stronger neural activation in response to negative emotional faces in the brain regions implicated in processing social rejection (i.e., dorsal ACC, anterior insula, Gillath et al., 2005) and regions implicated in threat detection (i.e., amygdala, Vrtička et al., 2008) when primed with neutral schema.

## Materials and Methods

### Participants

Our sample consisted of 42 right-handed undergraduate students (aged 18–27 years, mean 21.16 ± 1.57 years, 19 men) of a Chinese University, with normal or corrected-to-normal vision. All participants were in a romantic relationship for at least 6 months; subjects whose scores on the anxiety component of the Chinese version of the Experience in Close Relationships scale (ECR; Tonggui and Kazuo, 2006) were one standard deviation above or below average were invited to participate in the experiment. After excluding participants with incomplete experimental data, the final sample comprised 19 high-anxiety (*M* = 88.63, *SD* = 6.62) and 19 low-anxiety participants (*M* = 54.56, *SD* = 10.66). Exclusion criteria included having a current psychiatric diagnosis, current substance abuse, or a history of taking psychotropic medication within 2 weeks of the experiment. All participants provided written informed consent prior to the study. The study was approved by the Institutional Human Participants Review Board of Southwest University Imaging Center for Brain Research.

### Materials and Procedure

#### Materials

Participants were asked to provide their current partner’s full name in Chinese as priming materials in the secure priming condition. Acquainted names that matched the number and the frequency of words were used as the neutral prime materials to control for familiarity and friendly feelings accordingly. The target stimulus set consisted of 20 aversive and 20 happy facial expressions based on normative ratings from the Chinese Facial Affective Picture System (CFAPS). The photographs comprised 20 individuals (20 female) each exhibiting one of two expressions (happiness and disgust). The two groups of pictures were matched for general content, including color and size, and were balanced on gender and emotional arousal (*t*-test, *p* < 0.05).

#### Procedure

Prior to the experiment, participants completed the online ECR as a personality measure. The reliability and validity of the ECR scale has been repeatedly demonstrated for the Chinese population (Tonggui and Kazuo, 2006). The scale consisted of 36 items, with self-report measures to assess adult attachment styles in terms of two dimensions, with 18 items in each of the two dimensions – the attachment anxiety dimension (e.g., “I worry about losing the love of others”) and the attachment avoidance dimension (e.g., “I prefer not to show others how I feel deep down”). Participants were required to rate the extent to which they agree with each statement on a 7-point rating scale ranging from 1 = “not at all” to 7 = “very much.” Total scores were the sum of responses on items relevant to the anxious and avoidance dimensions, such that higher scores on each dimension indicated a less secure attachment style. In the current study, the Cronbach’s alpha coefficients for both dimensions were good (0.92 for the 18 attachment anxiety items and 0.85 for the 18 attachment avoidance items). Based on their anxiety scale scores, the 42 participants were divided into two groups [anxiety (AX) and secure (SE)] and were selected to participate in the fMRI experiments. Based on Fazio and Williams (1986) original procedures, we adopted a modified paradigm combining attachment secure priming with a dual-valence task. In this experiment, a secure based schema was activated (or primed) by presenting individuals with the name of a supportive person. In this study, the partner’s name was used as a secure prime. Corresponding to each of these presentations, a neutral name was used as a neutral prime (control condition). Each trial lasted for 7–8 s, and consisted of a fixation cross presented for 1000 ms, followed by a priming name (partner/neutral) presented on the center of the screen for 2000 ms, which was followed by a blank screen for 1000 ms; each prime was followed by a target face with positive or negative valence randomly selected from 20 positive and negative faces each. The target picture of an emotional face (positive/negative) lasted for 2000 ms. During this period, subjects had to evaluate the emotional face as expressing either a positive or a negative emotion by pressing one of two buttons (1, -1) as quickly and as accurately as possible. Finally, a blank screen was presented as a buffer, the duration of which was randomly chosen to be 1 s or 2 s. The task contained four conditions: (1) secure prime, positive face [secure positive (SP)], (2) secure prime, negative face [secure negative (SN)], (3) neutral prime, positive face [neutral positive (NP)], and (4) neutral prime, negative face [neutral negative (NN)]. Participants completed two rounds, each consisting of 80 trails with 20 trails in each of the four conditions. During the course of the experiment, subjects lay in the supine position in the MRI scanner with their hands holding a button box. Accuracy and reaction time measurements were collected while subjects performed the tasks in the scanner using the same software that was used for stimulus presentation (Presentation, Neurobehavioral Systems, Albany, CA, USA).

### Behavioral Data Analysis

All behavioral statistical analyses were performed using SPSS 16.0 (Statistical Packages for the Social Sciences, Version 16.0, SPSS Inc., USA) with a level of significance of *p* < 0.05. We performed a 2 × 2 × 2 repeated-measures ANOVA on the reaction times of evaluative responses, with the attachment style, priming type, and emotional valence as the factors. Trials that were incorrectly categorized, and trials with reaction times that were three standard deviations above or below the mean of each trial were excluded from statistical analyses. On an average, less than 5% of all trials were excluded from this experiment.

### fMRI Data Acquisition and Data Analysis

Brain images were acquired with a Siemens 3T scanner (Siemens Magnetom Trio TIM, Erlangen, Germany). An echo-planar imaging (EPI) sequence was used for collecting data of functional images, and 303 T2^∗^-weighted images were recorded per run [TR = 2000 ms; TE = 30 ms; in-plane resolution = 3.4 mm × 3.4 mm; flip angle = 90°; inter-slice skip = 0.99 mm; field of view (FOV) = 220 mm × 220 mm; voxel size = 3 mm × 3 mm × 3 mm; matrix = 64 × 64; 32 interleaved 3-mm thick slices]. T1-weighted images consisted of 176 slices that were 1 mm thick, with an in-plane resolution of 0.98 mm × 0.98 mm (TR = 1900 ms; TE = 2.52 ms; flip angle = 9°; FOV = 250 mm × 250 mm; voxel dimensions = 1 mm × 1 mm × 1 mm). SPM8 (Wellcome Department of Cognitive Neurology, London, UK) was used to preprocess the functional images (Friston et al., 1994) on the Matlab 6.5.1 platform (Mathworks Inc., Natick, MA, USA). Images were corrected for differences in slice timing and were then realigned to estimate and correct for the six parameters of head movement. The realigned images were then normalized to the Montreal Neurological Institute (MNI) space (3 mm × 3 mm × 3 mm) and spatially smoothed with a Gaussian kernel (FWHM = 6 mm × 6 mm × 6 mm).

After preprocessing, subjects with more than 2.5 mm motion across functional runs (*N* = 4) were excluded from the analysis and 38 participants [highly anxious individuals (*N* = 19), secure individuals (*N* = 19) (female *N* = 21, male *N* = 17)] were included in the analysis. For each subject, both the runs were modeled in one general linear model (GLM). We chose the presentation of the stimulus (prime name and response categories) as the onset time. The explanatory variables were convolved with the canonical hemodynamic response function, eight conditions (in ANOVA1) and four conditions (in ANOVA2) were used as regressors in the GLM, respectively, and the six realignment parameters for each subject were included as confounding factors in the GLM in both the ANOVAs.

### Statistical Image Analysis

Statistical analyses were performed on individual participants’ data with the GLM implemented in SPM8. Two levels of ANOVA were used to deal with fMRI data. At the first (subject) level, contrast maps were generated for each subject with each type of stimulus contrasted to the baseline of the average whole brain activity. As described above, besides the attachment style as a between-subject factor, there were two conditions in the priming interface, in addition to four combinations of stimulus variables defined in the response interface. The images from the priming interface were entered into a full-factorial 2 (attachment group) × 2 (prime type) ANOVA, with prime type (partner/neutral) as a within-subject factor and attachment style (high/low anxiety) as a between-subject factor. Similarly, the other full-factorial ANOVA consisted of the images in the response interface, with prime type (partner/neutral) and target valence (positive/negative) as within-subject factors and attachment style (high/low anxiety) as a between-subject factor. For the interaction analysis, the average percent signal change was extracted from the significant cluster for each condition using MarsBar (Brett et al., 2002) to examine the direction of the response; following this, the SPSS 16.0 was used to conduct a simple effect analysis. The statistical threshold used for these data was first set to *p* < 0.005 (one-tailed, uncorrected) at the individual voxel level. Then we performed AFNI’s AlphaSim program^1^ to correct for multiple comparisons. We ran 1000 Monte Carlo simulations with voxel-level *p* < 0.005, cluster size > 87, corresponding to a corrected *p* < 0.05 as determined by AlphaSim correction (Yang et al., 2016; Chen and Mo, 2017). The FWHM of the individual t-maps was used as the Gaussian filter, along with a cluster connection radius of 5 mm, as previous studies have demonstrated that 1000 simulations are adequate for an fMRI study (Brass et al., 2007; Chen et al., 2013). All coordinates have been reported using the MNI convention.

## Results

### Participant Characteristics

The demographic characteristics of the participants in this study are shown in **Table 1**. No significant differences were seen between the AX and SE groups in age, years of education, trait anxiety, and attachment avoidance. As expected, the AX group had significantly higher scores on the attachment anxiety than the SE group (*t* = 11.897, *p* < 0.001). At the time of assessment, all participants were involved in their current romantic relationship for an average of 18.2 months. The demographic characteristics of the subgroups are shown in **Table 1**.

**Table 1:** Demographic characteristics of highly attachment anxiety individuals and matched controls.

Variable	AX (*SD*) (*n* = 19)	*SE* (*SD*) (*n* = 19)
Age (years)	20.7 (1.2)	21.6 (1.5)
Female	12	9
Education (years)	14.2 (1.2)	14.6 (1.4)
AX scores in ECR	88.6 (6.6)	50.5 (8.4)
AV scores in ECR	45.2 (11.3)	45.4 (11.4)
Trait anxiety score	41.5 (9.1)	37.4 (4.6)
Length of relationship	17.3 (5.7)	18.8 (8.0)

*SD, standard deviation; ECR, Experience of Close Relationship scale; AX, attachment anxiety; AV, attachment avoidance.*

### Behavioral Data

**FIGURE 1:**
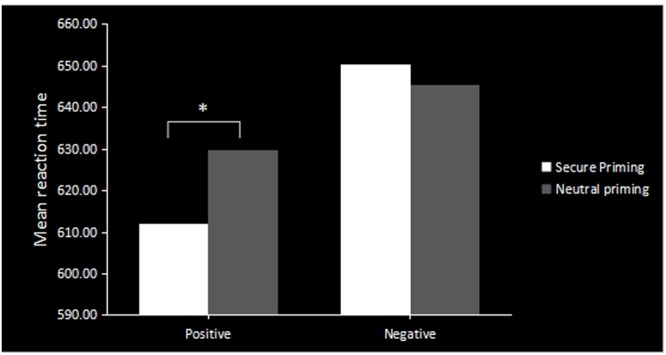
**The behavioral result of emotional process suggested faster response-time to positive faces in secure priming than neutral priming.** The asterisks (^∗^) indicate significant differences (^∗^*p* ≤ 0.05).

A 2 (secure/neutral prime) × 2 (positive/negative face) × 2 (high/low anxiety) repeated-measures ANOVA revealed a significant main effect of target valence [*F*(1,37) = 9.376, *p* < 0.01] as well as a significant prime type and target valence interaction [*F*(1,37) = 6.071, *p* < 0.05]. The simple effect analysis (**Figure 1**) suggested that participants responded faster to positive emotional faces in the secure prime condition than in the neutral prime condition [*F*(1,37) = 5.984, *p* < 0.05], which was in line with our assumptions. However, for negative faces, there was no significant difference in the response times between the secure prime and neutral prime conditions (*F* = 1.747, *p* = 0.194). There was no significant main effect of prime condition (*F* = 0.625, *p* = 0.434), target valence (*F* = 0.056, *p* = 0.814), attachment style (*F* = 0.062, *p* = 0.804) or any possible interaction on the accuracy of the responses.

### Imaging Data

#### Group Analysis of Attachment Secure Priming

**FIGURE 2:**
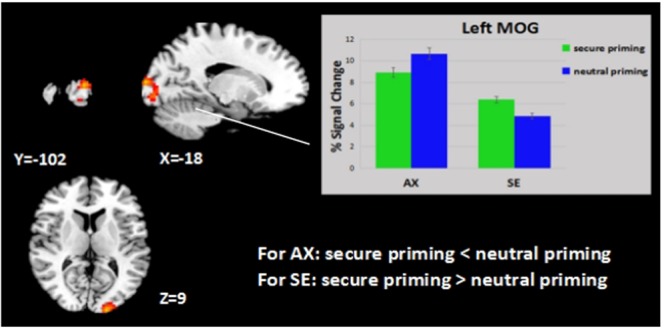
**Attachment group × priming effect.** Displayed is a rendering of (attachment group × priming) interaction from the factorial analysis. Bar chart displays the percent signal change in highly attachment anxiety and in controls for secure priming (green) and neutral priming (blue) conditions. Detailed results can be found in **Table 2B**.

**FIGURE 3:**
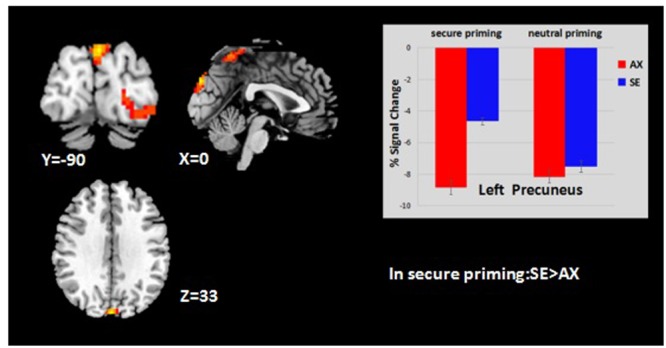
**Attachment group × priming effect.** Displayed is a rendering of (attachment group × priming) interaction from the factorial analysis. Bar chart displays the percent signal change in controls (blue) and in highly anxiety (red) for secure priming and neutral priming conditions. Detailed results can be found in **Table 2B**.

Our study combines two lines of research, the first involving behavioral differences in emotional processing in secure priming between anxious attached and secure attached groups, and second involving neural correlates of attachment secure priming and its effects on emotion processing. Although our primary interest was in examining the effects of attachment style on emotion processing, we began our analyses at the level of the entire group of 38 participants to see whether our secure priming-related findings were in line with findings from previous studies. When we compared the group effects of the secure priming and neutral priming conditions, the contrast (task > baseline) revealed a significant activation in the whole brain analysis. Both groups showed similar patterns of greater activation with secure priming than neutral priming (*p* < 0.05, Alphasim corrected, **Table 2**). An interaction effect of priming and attachment style emerged in two clusters [*p* < 0.05, Alphasim corrected; peak voxel coordinates: (-18, -102, 9) and (0, -90, 33); peak *Z* scores = 4.04 and 4.55]. The average percent signal change was extracted from both significant clusters to determine the nature of this interaction. The simple effect analysis indicated that the SE group showed higher activation (secure vs. neutral) in the left middle occipital gyrus (MOG/BA18) [*F*(1,37) = 12.484, *p* < 0.01], an area in which the AX group exhibited deactivation [*F*(1,37) = 15.965, *p* < 0.001] (see **Figure 2**). In addition, the same contrast (secure vs. neutral) revealed that the precuneus was significantly more activated in the SE group than in the AX group [*F*(1,37) = 4.408, *p* < 0.05] (see **Figure 3**).

**Table 2:** Peak coordinates from factorial analysis of priming type, attachment group and interactions. All activation peaks were assigned to the most probable brain areas as indicated by the SPM Anatomy Toolbox (Eickhoff et al., 2005, 2007).

Region	BA	MNI			*z*-Value	Volume (voxels)
		*x*	*y*	*z*		
**(A) Main effect of priming type (secure priming > neutral priming)**					
Pc/PCC	31	–6	–57	27	6.82	803
Left anterior cingulate	32/24	–3	33	–9	5.25	257
Left orbital frontal cortex	11	–3	51	–12	6.12	107
Left middle occipital gyrus	19	–45	–72	6	4.28	177
Left middle temporal gyrus	21	–63	–15	–18	5.25	274
Left temporal pole		–39	24	–21	5.12	209
**(B) Interaction: priming type × attachment group**					
Left middle occipital gyrus	18	–18	–102	9	4.04	153
Precuneus		0	–90	33	4.45	143

*Pc/PCC, the precuneus/posterior cingulate cortex.*

#### Group Analysis of Emotional Face Processing

As mentioned above, one of the primary interests of this study was to examine whether the effects of attachment secure priming on emotional information processing interact with attachment anxiety. In order to study this more closely, we first conducted a repeated measures ANOVA using images from both the groups. There was a significant main effect of priming (secure vs. neutral) in two clusters (Alphasim corrected, *p* < 0.05) (see **Table 3**). Both these regions showed a deactivation while viewing emotional faces following the secure prime condition, compared to the neutral prime condition. An interaction effect of priming, attachment style and valence emerged in two clusters [*p* < 0.005, *k* > 20, uncorrected; peak voxel coordinates: (-33,51,0) and (24,63,9); peak *Z* scores = 3.79 and 3.40].

**Table 3:** Peak coordinates from repeated measures analysis of variance and one sample *t*-test. All activation peaks were assigned to the most probable brain areas as indicated by the SPM Anatomy Toolbox (Eickhoff et al., 2005, 2007).

Region	BA	MNI			*z*-Value	Volume (voxels)
		*x*	*y*	*z*		
**(A) Priming contrast**					
(secure priming < neutral priming)						
Right precuneus	19	24	–78	33	4.68	206
Left cuneus	19	–15	–84	33	4.08	170
Right fusiform gyrus	37	30	–39	–18	4.53	92
(Interaction for three variables) attachment style^∗^priming^∗^valence						
Left middle frontal gyrus	10	–33	51	0	3.79	32
Right middle frontal gyrus	10	24	63	9	3.40	27
**(B) Priming contrast in AX during positive emotion processing**
(secure priming > neutral priming)
Right middle temporal gyrus	21	60	–27	–12	4.07	128
Right anterior cingulate Cortex	32	9	48	15	3.75	119
Left medial frontal gyrus	9	–6	54	39	4.27	352
**(C) Priming contrast in AX during negative emotion processing**
(secure priming < neutral priming)
Right fusiform gyrus	37	27	–42	–15	3.95	149
Left middle occipital gyrus	19	–39	–75	12	3.63	112
Right middle occipital gyrus	19	48	–75	12	3.78	239

#### Effect of Priming in Positive Emotion Processing

**FIGURE 4:**
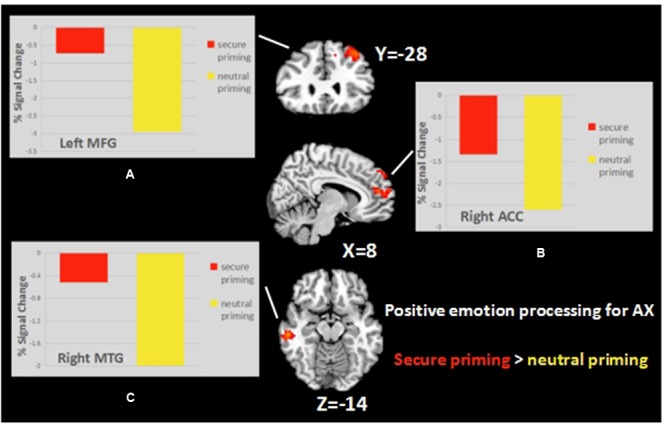
**The Secure priming > Neutral priming contrast in AX group revealed activation in the left MFG (A)**, right ACC **(B)**, and right MTG **(C)**.

To further understand the interaction of attachment secure priming and emotion processing, we explored the effect of the two priming conditions on emotion processing in a one-sample *t*-test individually for the two groups. In the AX group, three clusters (Alphasim corrected, *p* < 0.05) showed an effect of priming on the emotion recognition task. For all these regions (right middle temporal gyrus, the bilateral middle frontal gyrus, and the right anterior cingulate cortex), greater activation was seen while viewing positive emotional faces for the secure priming condition than the neutral priming condition (**Table 3B** and **Figure 4**). This effect was not seen in the SE group.

#### Effect of Priming in Negative Emotion Processing

**FIGURE 5:**
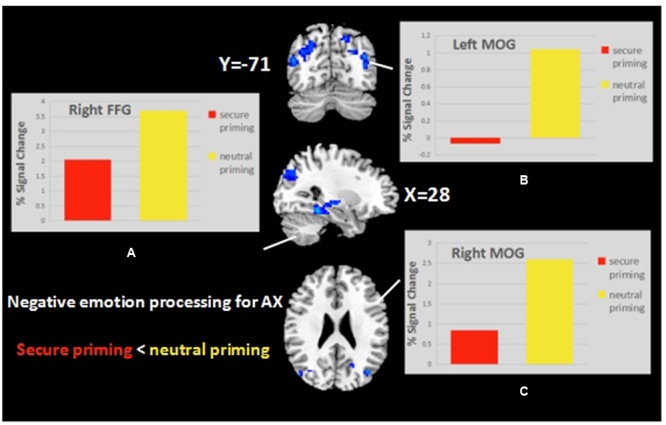
**The Secure priming < Neutral priming contrast in AX group revealed activation in the right FFG (A)** and the bilateral MOG **(B,C)**.

We hypothesized that participants with higher anxiety would have a different pattern of activation during negative emotion processing because, in behavioral studies, it was found that anxious subjects displayed hypervigilance in response to cues related to attachment threat or a prolonged overactivation of the attachment system. In the AX group, processing negative emotional faces in the attachment secure priming condition (compared to neutral priming) was associated with significant deactivation (Alphasim corrected, *p* < 0.05) in the three clusters located in the right fusiform gyrus, right parahippocampal gyrus, and bilateral middle occipital and middle temporal gyri (**Table 3C** and **Figure 5**). This effect was not seen in the SE group.

## Discussion

This study explored how chronic attachment orientations (attachment anxiety in the present study) mediate the interaction between attachment secure priming and emotional processing. The results showed that attachment secure priming interacted with attachment anxiety to affect the processing of emotional stimuli with both positive and negative valence. First, we combined both groups to examine the main effects of the two types of priming: secure priming, which was established by presenting cues characteristic of a secure attachment figure, and neutral priming, which was used as baseline to determine brain activations. Next, we conducted a whole brain comparison for the two emotional processing conditions, the main and interaction effects of which are discussed below. Finally, we compared the differential priming effects on processing facial expressions for both positive and negative emotional valence individually.

### The Main Effect of Attachment Secure Priming

For almost everyone, romantic love is a highly rewarding experience, which serves a crucial evolutionary purpose, namely, the maintenance and perpetuation of the species. Attachment security can be bolstered by presenting cues characteristic of a secure attachment figure, which is associated with the activation of dopamine-rich areas associated with the mammalian reward and motivation circuitry (Aron et al., 2005). Consistent with our expectations, the results of our group-level analyses revealed that attachment secure priming was associated with stronger activation in a large number of brain regions. The present finding of higher activation in the left-hemisphere in the secure priming rather than the neutral priming condition reflected a predominance of the left side (Lee et al., 2004). This is consistent with predictions that the strengthening of attachment security can trigger a positive emotional state, which has been demonstrated through the activation in the MTG, an area known to be associated with the priming effect (Shelton and Martin, 1992). Repeated secure priming in the current study was associated with the experience of positive emotions and increased activation of dopamine-rich brain areas such as the left OFG (Haber and Knutson, 2010). The brain regions that are activated significantly more in the secure priming than in the neutral priming condition, including the left temporal pole, ACC, MOG, MTG, and PCC are known neural substrates for romantic love. For example, Bartels and Zeki (2000) reported that adults activated the ACC while viewing pictures of their partners (Lane et al., 1997). As part of the anterior attention network (AAN), this region regulates the expectation and motivation of attention and is a critical region for emotional arousal. In addition, the MTG is known to be associated with priming effect, the PCC, with retrieval of happy memories (Immordino-Yang et al., 2009), and the MOG, with visual resource allocation. Our results show that presenting cues characteristic of an attachment figure effectively activates the secure base schema and induces a spreading in activation of certain brain areas, stronger emotional arousal, and positive memory retrieval, which reflect in the data as shorter response times to expressions that reveal emotional valence. The SE group showed increased activation in BA18 related to secure priming, while the AX group showed reduced activation in the same area. This is likely to represent increased visual-information processing for secure priming in the SE group, but not in the AX group. Furthermore, a higher activation in the precuneus in the SE group than the AX group with the secure priming condition reflects a stronger ability to integrate information.

### Effect of Secure Priming on Facial Expression Processing

Analysis of main effects identified deactivation in the right fusiform gyrus and bilateral parietal-occipital cortex in the secure priming condition, but not in the neutral priming condition. These areas are thought to be associated with negative emotion processing (Heller et al., 2003), and the right fusiform gyrus is considered to be critical in processing cues that are above conscious level of perception (supraliminal cues). Moreover, as the fusiform gyrus is closely related to attentional bias and detection of emotional information (Amin et al., 2004), the higher one’s detection level reaches, the stronger the activation in the fusiform gyrus is likely to be.

The reduced activation in the fusiform gyrus in the AX group in our study suggests that individuals are less engaged in emotion recognition in secure priming, with fewer cognitive resources recruited. This, along with the higher activation seen before the presentation of emotional facial stimuli, implies that cues related to a secure figure can capture the attention resources, thereby affecting the performance in subsequent cognitive tasks. More importantly, this process may interfere with the employment of the overactivation strategy in anxious individuals as we predicted. These findings cannot be attributed to attachment avoidance or trait anxiety, since both of these factors were controlled. However, whether attachment anxiety modulates the effects of priming on emotion processing still remains to be verified. Although the triple interaction effect was not significant, the complex relationships between variables can be further delved into by creating contrasts between conditions within individual groups.

### Interaction of Secure Priming with Chronic Attachment Anxiety in Emotion Processing

The results from the one-sample *t*-test showed that chronically insecure-anxious attachment orientations interacted with attachment security priming to affect the processing of both, positive and negative information. Even though the behavioral data suggest that secure priming can facilitate positive emotion processing and hinder negative emotion processing, the difference in neural activation between the priming conditions appeared only in the AX group, indicating that attachment anxiety may be the moderating variable. Notably, the patterns of emotional information processing for the attachment anxious and their secure counterparts were different, depending on the experimental condition. Thus, when attachment security was activated, the SE group did not show any significant increase or decrease in activation related to secure priming during both positive and negative emotion processing. In contrast, positive emotion processing in the AX group was associated with activation in the right MTG, the right MPFC, and the left DLPFC in the secure priming condition; greater activation in the right MTG confirmed the effect of secure priming on positive emotion processing in anxious individuals. Interestingly, participants with high chronic anxiety turned their attention toward the positive emotion under the effect of secure priming, which is called the “mood-congruency effect.” On the other hand, for the negative emotion, the appraisal of negative social cues was primarily related to attachment anxiety dimension, consistent with the assumptions of attachment theory (Mikulincer and Shaver, 2007). Our results suggest that high anxiety was associated with deactivation in the right fusiform gyrus, right parahippocampal gyrus, and bilateral MOG, all known to be related to appraisal of potential threats concerning information related to immediate danger for survival in humans. Thus, a set of regions typically associated with negative affect were seen to be activated when the subjects were presented with unpleasant facial expressions; however, this social aversion component can be modulated by a secure attachment style (Coan et al., 2006). More importantly, our results demonstrate an interaction between dispositional attachment insecurities and attachment security that was temporarily activated to explore the link with emotion processing (Carnelley and Rowe, 2010). In a review of literature relevant to repeated attachment secure priming, researchers expressed reservations regarding the effects of attachment secure priming being independent of dispositional attachment insecurities (Gillath et al., 2008); this suggestion is based on a research (Mikulincer et al., 2002) which reported a clear interaction between the subliminal activation of the attachment system (by presenting the word “abandonment”) and chronic attachment orientations. In addition, there are recent studies describing the interactions between experimentally induced security and dispositional attachment style which affect the way in which individuals deal with painful emotions (Cassidy et al., 2009) and feelings of regret associated with mistakes in the past (Schoemann et al., 2012). The use of the partner’s name in our study additionally led to a weaker response in regions responsible for negative emotion processing and reflection in the AX group, with fewer cognitive resources devoted. These findings are consistent with our hypotheses that the activation of attachment security may bring beneficial effects to emotional information processing regardless of variations in the expression of chronic attachment anxiety. Individuals with higher anxiety levels were seen to be more sensitive to information related to immediate threats in the current environment; however, the brain systems mediating this response are no longer recruited when these individuals are presented with cues related to their partners. In other words, the secure priming might be a dynamic balance or a “push-pull” mechanism for individuals with high anxiety. More specifically, activating a sense of attachment security by priming the secure base schema results in decreased activation in regions associated with negative emotions. Given that the consequences of raising security activation on emotion regulation (e.g., Shaver et al., 2009) are mediated mostly through cognitive processes, we can expect that priming the secure base schema should have beneficial effects on emotion processing for individuals with attachment anxiety.

## Conclusion

The current study investigated the priming effects of secure base schema on processing of facial expressions. Our results provide evidence of a direct effect of security priming on emotional processing, moderated by participants’ chronic attachment anxiety. More importantly, this process may interfere with the employment of the overactivation strategy in anxious individuals. The results highlight the beneficial effects of secure priming in individuals with chronically insecure attachments. Theoretically, the results are also consistent with findings of recent studies (e.g., Kafetsios et al., 2014) that highlight the dynamic nature of attachment organization in the context of emotional information processing.

## Author Contributions

XC and QT designed experiments. QT and JH carried out experiments. YL analyzed experimental results. QT wrote the manuscript.

## Conflict of Interest Statement

The authors declare that the research was conducted in the absence of any commercial or financial relationships that could be construed as a potential conflict of interest.

## Footnotes

1 http://afni.nimh.nih.gov/pub/dist/doc/manual/AlphaSim.pdf
